# Dr. Richardson on Chloral Hydrate

**Published:** 1871-03

**Authors:** 


### ARTICLE V.

### *Dr. Richardson on Chloral Hydrate.*

Before commencing his lecture on Experimental and Practical Medicine on Tuesday last, Dr. Richardson offered some observations on the subject of Hydrate of Chloral to which we would call the attention of our readers. He said:

My lecture to-day is on Suspended Animation, but I will ask you to allow me first to offer a note or two on another subject, at this moment of urgent importance. I refer to the administration of the hydrate of chloral. There have recently been two assured deaths from hydrate. In the course of the past ten days, I have myself been consulted not fewer than three times on what have been considered dangers attending the administration of the hydrate, and I know generally that doubt and uneasiness prevail in the profession respecting the abuse as opposed to the use of this agent. I think it right, therefore, as I had much to do in introducing hydrate of chloral into medical practice in England, to answer a few of the questions that are most pressing from this place, where so many demonstrations of the action of the hydrate have been carried out.

1. *Is the practice of resorting to the use of hydrate of chloral, as a narcotic, in the absence of medical advice and*

*direction becoming a common practice among the people?* The answer to this question is strictly affirmative. The novelty of its administration and of proving its effects at an end, the hydrate is not at the present time used so largely by the medical profession as it was a few months ago, when its true place in the Materia Medica was less clearly defined. The sale of the hydrate of chloral to medical men is consequently considered as declining, while the general sale is, perhaps, increasing. Corresponding with this state of things we, in in the profession, are becoming conversant with cases of what may not improperly be called "chloral drinking," and in which serious and singular symptoms are presented. Three classes of people specially resort to hydrate of chloral —viz., alcoholic devotees, who take the substance to counteract excess of alcohol and to relieve alcoholic delirium; sufferers from neuralgia and other painful chronic diseases, who find in the substance temporary relief from pain; and persons having much mental worry, grief, or care, who, flying to it at first in order to obtain sleep, continue it until the occasional practice becomes a persistent habit. As an indication of the quantity of hydrate of chloral used in this country since its introduction here about a year and a half ago, I may state, incidentally, on what I have every reason to consider reliable authority, that one commercial house alone has supplied the English drug market with ten tons of the substance; three other houses have, it is supposed, supplied as much; so that fifty tons weight have been, on this calculation, sent out—an amount which, divided into grains, would yield over thirty-six millions of narcotic doses to England alone since August, 1869.

2. *What is a dangerous and what is a fatal single dose of hydrate of chloral?* The largest dose I have known to be taken is one hundred and twenty grains. This dose produced a prolonged and dangerous coma, but recovery ultimately followed. I think we may consider a hundred and twenty grains, as a maximum dose for an adult, dangerous, but not of necessity fatal. Beyond a hundred and twenty grains the danger increases, and a hundred and eighty grains may be considered a dose that would prove, in the majority of cases, positively fatal.

3. *What quantity of hydrate of chloral can be given with safety in divided doses during a stated period of time, say of twenty-four hours?* Judging from the physiological effects of hydrate of chloral in relation to dose, and to order of phenomena in relation to time, I should infer that the body cannot decompose and throw off the hydrate more rapidly than at the rate of from five to seven grains per hour. There will be difference according to age of person, the temperature to which the body is exposed after the dose has been taken, and the largeness of the dose, a small dose being disposed of quicker in proportion than a large one. But the variation is not such as to alter materially the rate of action from the estimate given. I should consider, consequently, that a hundred and twenty grains, administered, even in divided doses, in twenty-four hours, would be the safe limit of administration. In the treatment of tetanus this proportion has been exceeded, but not, I think, to the safety of the patient; for the fact that the hydrate of chloral overcomes or reduces the spasm is no safeguard against its own poisonous effects. From what I know, I conclude that the hydrate of chloral can be given to the extent of overcoming the severest spasm; but if the dose be carried too far, with the determination of removing spasm at all risks, the success may easily be bought at the expense of a fatal narcotism from the remedy.

4. *Does the frequent administration of hydrate of chloral lessen or increase the danger of the administration?* On this question I am forced to state the frequent administration of the hydrate of chloral, though it may suggest greater confidence in it on the part of those who take it, increases the danger from an excessive dose. Hydrate of chloral differs from opium in this respect. Opium produces chronic symptoms peculiar to itself, but the dose may be steadily increased without any immediate danger from the increase.

Hydrate of chloral cannot be used in this accumulative way without danger. In a word, although a person may become habituated to hydrate of chloral, there is a limitation of the quantity to be taken safely, which limitation is not materially modified by persistence in the habit of taking it, but rather the reverse.

After discussing three other questions, relating to the symptoms and pathological changes incident to the habitual use of chloral hydrate, to the chemical tests for the hydrate in cases where it has caused death, and to the post-mortem distinctions in instances of chronic poisoning by the hydrate and by chloroform, Dr. Richardson concluded by observing that as the world was indebted to the profession of medicine for the benefits derivable from the hydrate of chloral, it behoved the members of the profession to use their influence in protecting the public from an agent which, under improper use, might be turned from its good purpose to positive evil.—*London Lancet.*

---

